# A Cross-Domain Collaborative Filtering Algorithm Based on Feature Construction and Locally Weighted Linear Regression

**DOI:** 10.1155/2018/1425365

**Published:** 2018-02-12

**Authors:** Xu Yu, Jun-yu Lin, Feng Jiang, Jun-wei Du, Ji-zhong Han

**Affiliations:** ^1^School of Information Science and Technology, Qingdao University of Science and Technology, Qingdao 266061, China; ^2^Institute of Information Engineering, CAS, Beijing 100093, China

## Abstract

Cross-domain collaborative filtering (CDCF) solves the sparsity problem by transferring rating knowledge from auxiliary domains. Obviously, different auxiliary domains have different importance to the target domain. However, previous works cannot evaluate effectively the significance of different auxiliary domains. To overcome this drawback, we propose a cross-domain collaborative filtering algorithm based on Feature Construction and Locally Weighted Linear Regression (FCLWLR). We first construct features in different domains and use these features to represent different auxiliary domains. Thus the weight computation across different domains can be converted as the weight computation across different features. Then we combine the features in the target domain and in the auxiliary domains together and convert the cross-domain recommendation problem into a regression problem. Finally, we employ a Locally Weighted Linear Regression (LWLR) model to solve the regression problem. As LWLR is a nonparametric regression method, it can effectively avoid underfitting or overfitting problem occurring in parametric regression methods. We conduct extensive experiments to show that the proposed FCLWLR algorithm is effective in addressing the data sparsity problem by transferring the useful knowledge from the auxiliary domains, as compared to many state-of-the-art single-domain or cross-domain CF methods.

## 1. Introduction

The rapid growth of the information on the Internet demands intelligent information agent that can sift through all the available information and find out the most valuable to us. In recent years, recommender systems [1, 2] are widely used in e-commerce sites and online social media and the majority of them offer recommendations for items belonging to a single domain. Collaborative filtering (CF) algorithms [3] are the most widely used methods for recommender systems and they can be categorized into three classes, including memory-based algorithms [4], model-based algorithms [5], and matrix factorization based algorithms [6].

However, in real-world recommender systems, users usually dislike rating items and the items rated are very limited. Thus the rating matrix is very sparse. The sparsity problem has become a major bottleneck for most CF methods. To alleviate this difficulty, recently a number of cross-domain collaborative filtering (CDCF) methods have been proposed [7]. CDCF methods exploit knowledge from auxiliary domains (e.g., movies) containing additional user preference data to improve recommendation on a target domain (e.g., books) containing less user preference data. They can effectively relieve the sparsity problem in the target domain.

Currently, CDCF methods can be categorized into two classes. One class [8–11] assumes shared users or items. This assumption commonly appears in the real world. For instance, Amazon website contains different domains, including Books, Music CDs, DVDs, and Video tapes. They share the same user set though their items are totally different. For another instance, Amazon Book Network and Dang-Dang Book Network sell similar products to different users. It is easy to find an intersection in which the two domains share the same items. The other class contains a limited number of CDCF methods [12, 13] that do not require shared users and items. However, they assume that both users and items in an auxiliary data source are related to the target data.

Among previous works of the first class, Berkovsky et al. [8] mention a neighborhood based CDCF (N-CDCF), which can be viewed as the cross-domain version of a memory-based method, that is, N-CF [4]. Hu et al. [9] mention a matrix factorization based CDCF (MF-CDCF), which can be viewed as the cross-domain version of a matrix factorization based method. Both N-CDCF and MF-CDCF accommodate items from all domains into a single matrix so as to employ single-domain CF methods. However, they assume the homogeneity of items. Obviously, items in different domains may be quite heterogeneous, and the above two models fail to take this fact into account.

Singh and Gordon [10] propose a Collective Matrix Factorization (CMF) model. CMF couples rating matrices for all domains on the* User* dimension so as to transfer knowledge through the common user-factor matrix. Hu et al. [9] propose a generalized Cross-Domain Triadic Factorization (CDTF) model over the triadic relation user-item-domain. Considering that not all the auxiliary domains are equally correlated with the target domain, CMF and CDTF assign different weights for different auxiliary domains. This is an advantage of them over N-CDCF and MF-CDCF. However, CMF does not provide a mechanism to find an optimal weights assignment for the auxiliary domains. Though CDTF assigns the weights based on genetic algorithm (GA), the performance is susceptible to the setting of the initial population.

Pan et al. [11] propose a Transfer by Collective Factorization (TCF) model. TCF model requires that the target domain and the auxiliary domain share the same aligned users and items simultaneously. In this assumption, they explore how to take advantage of knowledge in the form of binary ratings (like and dislike) to alleviate the sparsity problem in numerical ratings. The two-side (user-side and item-side) assumption can provide more precise information on the mapping between auxiliary and target data, which can lead to higher performance. However, this assumption does not very commonly appear in the real world.

Among previous works of the second class, Li et al. [12] propose a codebook-based knowledge transfer (CBT) for recommender systems. CBT achieves knowledge transfer with the assumption that both auxiliary and target data share the cluster-level rating patterns (codebook). Further, Li et al. [13] propose a rating-matrix generative model (RMGM). RMGM is derived and extended from the Flexible Mixture Model (FMM) [5], and we can consider RMGM as a multitask learning (MTL) [14] version of CBT with the same assumption. Both CBT and RMGM require two rating matrices to share the cluster-level rating patterns. In addition, CBT and RMGM cannot make use of user- or item-side shared information.

In this paper, we assume the auxiliary domains contain dense rating data and share the same aligned users with the target domain. Previous works on this assumption cannot compute proper weights for different auxiliary domains. In order to overcome this drawback, we propose a cross-domain collaborative filtering algorithm based on Feature Construction and Locally Weighted Linear Regression (FCLWLR). We first construct features both in the target domain and in the auxiliary domain. We use different features to represent different domains. Instead of assigning proper weights to different auxiliary domains, we just assign proper weights to different features. Then we combine all the features together and convert the cross-domain recommendation problem into a regression problem. Therefore, the important information in the auxiliary domains can be transferred to the target domain by the constructed features from the auxiliary domains. Finally, a nonparametric regression method, that is, Locally Weighted Linear Regression (LWLR) model [15], is used to solve the regression problem. We conduct extensive experiments to show that the proposed algorithm can outperform many state-of-the-art single-domain or cross-domain CF methods.

The remainder of this paper is organized as follows: Section 2 reviews the related works on CDCF methods.  Section 3 proposes our FCLWLR model. In Section 4, we conduct extensive experiments to test the performance of the proposed algorithm. We conclude the paper and give future works in Section 5.

## 2. Related Works

Some of the earliest work on CDCF was carried out by Berkovsky et al. [8], who deployed several mediation approaches for importing and aggregating user rating vectors from different domains. Currently, CDCF methods can be categorized into two classes. One class assumes shared users or items [8–11], and the other class does not require shared users or items in different domains [12, 13].

In the first class, Berkovsky et al. [8] mention an early neighborhood based CDCF* (N-CDCF)*. As neighborhood based CF (N-CF) computes similarity between users or items, which can be subdivided into two types, user-based nearest neighbor (N-CF-U) and item-based nearest neighbor (N-CF-I), the N-CDCF algorithm can also be divided into two types: a user-based neighborhood CDCF model (N-CDCF-U) and an item-based neighborhood CDCF model (N-CDCF-I). For simplicity, we only give a detailed review on N-CDCF-U, and the detailed method of N-CDCF-I is in the same manner.

Let **D** = {*D*_0_, *D*_1_,…, *D*_*m*_} denote all the domains for modeling, *U* = {*u*_1_, *u*_2_,…, *u*_*n*_} denote the users in **D**, and *I*_*k*_ = {*i*_1_^*k*^, *i*_2_^*k*^,…, *i*_*n*(*k*)_^*k*^} denote items belonging to the domain *D*_*k*_  (0 ≤ *k* ≤ *m*), where *n*(*k*) denotes the item set size of *D*_*k*_. For a user-based CDCF algorithm, we first calculate the similarity, *s*_*u*,*v*_, between the users *u* and *v* who have corated the same set of items. The similarity can be measured by the Pearson correlation:

(1) $$\begin{array}{c} s_{u,v}=\frac{\sum_{i\in i_{u,v}}\left(r_{u,i}-{\overset{-}{r}}_{u}\right)\left(r_{v,i}-{\overset{-}{r}}_{v}\right)}{\sqrt{\sum_{i\in i_{u,v}}{\left(r_{u,i}-{\overset{-}{r}}_{u}\right)}^{2}\sum_{i\in i_{u,v}}{\left(r_{v,i}-{\overset{-}{r}}_{v}\right)}^{2}}}, \end{array}$$

where *i*_*u*,*v*_ = *i*_*u*_∩*i*_*v*_  (*i*_*u*_ = ⋃_*d*∈*D*_*i*_*u*_^*d*^,  *i*_*v*_ = ⋃_*d*∈*D*_*i*_*v*_^*d*^) denotes the items over all domains **D** corated by *u* and *v*; *r*_*u*,*i*_ and *r*_*v*,*i*_ are the ratings on item *i* given by users *u* and *v*, respectively; and ${\overset{-}{r}}_{u}$ and ${\overset{-}{r}}_{v}$ are the average ratings of users *u* and *v* for all the items rated, respectively. Then the predicted rating of an item *p* for user *u* can be calculated by a weighted average strategy [4]:

(2) $$\begin{array}{c} {\overset{⌢}{r}}_{u,p}={\overset{-}{r}}_{u}+\frac{\sum_{v\in U_{u,p}^{k}}s_{u,v}\left(r_{v,p}-{\overset{-}{r}}_{v}\right)}{\sum_{v\in U_{u,p}^{k}}\left|s_{u,v}\right|}, \end{array}$$

where *U*_*u*,*p*_^*k*^ denotes the set of top *k* users (*k* neighbors) that are most similar to user *u* who rated item *p*.

**Figure 1:**
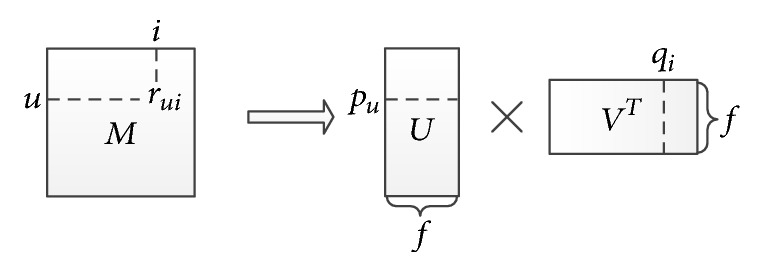
The Funk-SVD decomposition model.

In addition to the above model, the traditional MF model can also be employed to solve the CDCF problems straightforward. The Funk-SVD model is the most commonly used MF model [6]. As shown in Figure 1, for a single-domain collaborative filtering recommendation system, the Funk-SVD model maps both users and items to a joint latent factor space of dimensionality *f*.

In this model, each item *i* is associated with a latent vector *q*_*i*_ ∈ *R*^*f*^, and each user *u* is associated with a latent vector *p*_*u*_ ∈ *R*^*f*^. *q*_*i*_ measures the distribution of item *i* on those latent factors, and *p*_*u*_ measures the interest distribution of user *u* on those latent factors. The resulting dot product, *q*_*i*_^*T*^*p*_*u*_, captures the interaction between user *u* and item *i*. This approximates user *u*'s rating on item *i*, which is denoted by ${\hat{r}}_{ui}$ in the following form:

(3) $$\begin{array}{c} {\hat{r}}_{ui}=q_{i}^{T}p_{u}. \end{array}$$

To learn the latent vectors (*p*_*u*_ and *q*_*i*_), the Funk-SVD model minimizes the regularized squared error on the set of known ratings

(4) minq∗,p∗ ∑u,i∈κrui−qiTpu2+λqi2+pu2.

Here, *κ* is the set of the (*u*, *i*) pairs for which *r*_*ui*_ is known. The constant *λ* controls the extent of regularization to avoid overfitting and is usually determined by cross-validation [16]. An effective approach to minimize optimization problem (4) is stochastic gradient descent, which loops through all ratings in the training set. For each given training case, the system predicts *r*_*ui*_ and computes the associated prediction error

(5) $$\begin{array}{c} e_{ui}\overset{def}{=}r_{ui}-q_{i}^{T}p_{u}. \end{array}$$

Then it modifies the parameters by a magnitude proportional to *γ* (i.e., the learning rate) in the opposite direction of the gradient, yielding

(6) qi⟵qi+γeuipu−λqi,pu⟵pu+γeuiqi−λpu.

**Figure 2:**
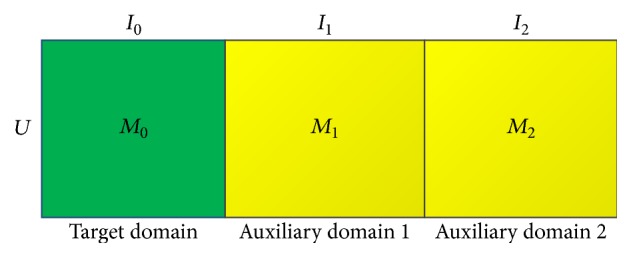
Horizontal concatenation of matrices for all domains.

Based on the traditional MF model, we can solve the CDCF problems straightforward. We can pour all the items from different domains together and then an augmented rating matrix, **M**_**D**_, can be built by horizontally concatenating all matrices as shown in Figure 2.

Thus we can use MF model to obtain the latent user factors and latent item factors. These latent factors are used for prediction. In this paper, the MF model on CDCF problems is denoted as MF-CDCF.

N-CDCF and MF-CDCF are developed straightforward from single-domain neighborhood and MF based CF methods, respectively. However, single-domain model assumes the homogeneity of items. Obviously, items in different domains may be quite heterogeneous, so N-CDCF and MF-CDCF fail to take this fact into account. Hence, the performance of them is not always satisfactory.

Singh and Gordon [10] propose the Collective Matrix Factorization (CMF) model. CMF [10] is proposed to collectively factorize one user-item rating matrix **R** ∈ *ℝ*^*n*×*m*^, **Y**⊙**R** ~ **UV**^*T*^, and one item-content matrix $\ddot{R}\in {\mathbb{R}}^{\ddot{n}\times m}$, $\ddot{R}\sim \ddot{U}{\ddot{V}}^{T}$, with the idea of sharing the same item-specific latent features **V**:

(7) $$\begin{array}{c} V=\ddot{V} \end{array}$$

which means that the item-specific latent feature matrix $\ddot{V}$ is shared as a bridge to enable knowledge transfer between two data sets.

Hu et al. [9] propose the CDTF model, in which they consider the full triadic relation user-item-domain to effectively exploit user preferences on items within different domains. They represent the user-item-domain interaction with a tensor of order three and adopt a tensor factorization model to factorize users, items, and domains into latent feature vectors. The rating of a user for an item in a domain is calculated by element-wise product of user, item, and domain latent factors. A major problem of tensor factorization, however, is that the time complexity of this approach is exponential as it is **O**(*k*^*m*^), where *k* is the number of factors and *m* is the number of domains. In addition, both CMF and CDTF need to adjust the weights of the auxiliary domains according to the similarities between each auxiliary domain and the target domain. Usually, computing proper weights is a tough problem.

Pan et al. [11] present a Transfer by Collective Factorization (TCF) model to transfer knowledge from auxiliary data of explicit binary ratings (like and dislike), which alleviates the data sparsity problem in numerical ratings. TCF collectively factorizes a 5-star numerical target data **R** and a binary like/dislike auxiliary data and assumes that both user-specific and item-specific latent feature matrices are the same. Besides the shared latent features, TCF uses two inner matrices to capture the data-dependent information, which is different from the inner matrix used in CBT [12] and RMGM [13]. TCF requires users and items of the target rating matrix and the auxiliary like/dislike matrix to be both aligned. In addition, they can only deal with the scenario of one auxiliary domain. Hence, it is not applicable to the problem studied in this paper.

In the second class, Li et al. [12] propose a CBT model. They first compress the auxiliary rating matrix, $\bar{R}\in {\mathbb{R}}^{\bar{n}\times \bar{m}}$, into an informative and yet compact cluster-level rating pattern representation referred to as a codebook, denoted as $\bar{B}\in {\mathbb{R}}^{d\times d}$. Then, they reconstruct the target rating matrix via codebook expansion **UBV**^*T*^ with the following constraint:

(8) $$\begin{array}{c} B=\bar{B} \end{array}$$

which means that the rating pattern is shared between target data and auxiliary data. Note that **U** ∈ {0,1}^*n*×*d*^ and **V** ∈ {0,1}^*m*×*d*^ are membership indicator matrices.

Further, Li et al. [13] propose a RMGM model. In this model, the knowledge is shared in the form of a latent cluster-level rating model. Each rating matrix can thus be viewed as drawing a set of users and items from the user-item joint mixture model as well as drawing the corresponding ratings from the cluster-level rating model. RMGM is a MTL version of CBT with the same assumption. Both CBT and RMGM require two rating matrices to share the cluster-level rating patterns. They assume that the items in an auxiliary data source (e.g., books) are related to the target data (e.g., movies). Hence they are also not applicable to the scenario studied in this paper.

## 3. Our Model

Since previous CDCF works cannot assign proper weights to different auxiliary domains, the recommendation performance is not always satisfactory. To overcome this drawback, in this paper, we first construct features in different domains and use the features to represent different domains. Then we combine the constructed features together and convert the original recommendation problem into a regression problem. Instead of assigning proper weights to different auxiliary domains, our aim is to assign proper weights to different features. In order to guarantee the accuracy of the weights for different features, we employ a nonparametric regression method, that is, Locally Weighted Linear Regression (LWLR) model, to solve the regression problem. Below we give the details of our model.

### 3.1. Feature Construction

Assume *D*_1_ is the target domain and *U*_1_ and *I*_1_ are the sets of users and items in domain *D*_1_. We use the trivial location information as the feature vector in the target domain. For example, we represent each user-item interaction (*u*, *i*, *r*) ∈ *U*_1_ × *I*_1_ × {1,2, 3,4, 5} in the target domain with a feature vector (*L*_*u*_, *L*_*i*_), where *L*_*u*_ and *L*_*i*_ denote the location information of user *u* and item *i*, respectively. However, such a two-dimensional feature vector is not sufficient to discriminate the user ratings. Hence, we require some other features to reflect the user preferences with the help of rating information from the auxiliary domains.

In this paper, we assume the auxiliary domains contain dense rating data and share the same aligned users with the target domain. In this scenario, we employ a user-based nearest neighbor (N-CF-U) algorithm to fill the missing ratings in the auxiliary domains. We expand the trivial location feature vector in the target domain with the corresponding row vectors from all the auxiliary domains. Thus we can effectively add more features to reflect the user preferences. Given a user-item interaction (*u*, *i*, *r*) ∈ *U*_1_ × *I*_1_ × {1,2, 3,4, 5} in the target domain, we can expand the location feature vector (*L*_*u*_, *L*_*i*_) in the target domain with all the row vectors of user *u* from all the auxiliary domains. Thus the expanded feature vector corresponding to the user-item interaction can be represented as (*L*_*u*_, *L*_*i*_, *r*_*u*_^1^,…, *r*_*u*_^*m*^), where *r*_*u*_^*i*^  (*i* = 1,…, *m*) represents the complete row vector of user *u* in the *i*th auxiliary domain obtained by N-CF-U algorithm.

### 3.2. Regression Model Building

Assume *D*_1_ is the target domain and *D*_2_,…, *D*_*m*+1_ denote the auxiliary domains. We can model the standard recommendation problem in the target domain *D*_1_ by a target function *y* : *F*_1_ × *F*_2_ × ⋯×*F*_*m*+1_ → *R*, where *F*_1_ denotes the feature vector (i.e., location information) in the target domain, *F*_*i*_  (2 ≤ *i* ≤ *m* + 1) denotes the feature vector (i.e., the corresponding row vector) in the (*i* − 1)-th auxiliary domain, and *R* denotes the rating value.

**Figure 3:**
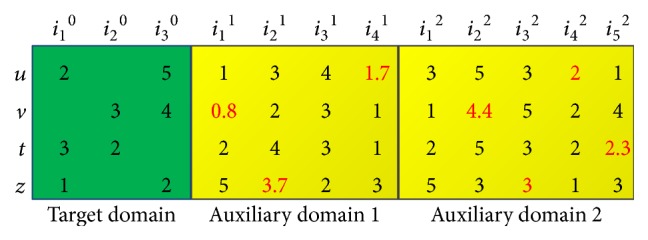
An illustration of cross-domain recommender system.

For example, we represent each user-item interaction (*u*, *i*, *r*) ∈ *U*_1_ × *I*_1_ × {1,2, 3,4, 5} with a feature vector (*L*_*u*_, *L*_*i*_, *r*_*u*_^1^,…, *r*_*u*_^*m*^) and a regression function value *r*. Thus we can represent each user-item interaction as a training sample, and the original recommendation problem can be converted into a regression problem. We use Figure 3 to illustrate our method.

In Figure 3, *u*, *v*, *t*, and *z* denote four users in all the domains, *i*_1_^0^, *i*_2_^0^, and *i*_3_^0^ denote three items in the target domain, *i*_1_^1^, *i*_2_^1^, *i*_3_^1^, and *i*_4_^1^ denote four items in the first auxiliary domain, and *i*_1_^2^, *i*_2_^2^, *i*_3_^2^, *i*_4_^2^, and *i*_5_^2^ denote five items in the second auxiliary domain. Firstly, we use N-CF-U algorithm to fill all the missing ratings which are marked in red color. Then we use 1, 2, 3, and 4 to denote the location of *u*, *v*, *t*, and *z*, and use 1, 2, and 3 to denote the location of *i*_1_^0^, *i*_2_^0^, and *i*_3_^0^. Thus the feature vector corresponding to the user-item interaction (*u*, *i*_1_^0^, 2) can be represented as

(9) 1,1︸features in the target domain,1,3,4,1.7︸features in Auxiliary domain 1,3,5,3,2,1︸features in Auxiliary domain 2.

The rating value 2 can be regarded as the regression function value. In the same manner, we can also represent other user-item interactions as training samples. Thus the rating matrix can be converted into a training set and we can convert the recommendation problem into a regression problem.

### 3.3. Regression Model Solving

In the constructed regression problem, the dimension of the feature vector is *s*_2_ + *s*_3_ + ⋯+*s*_*m*+1_ + 2, where *s*_*i*_  (2 ≤ *i* ≤ *m* + 1) is the item size of the (*i* − 1)th auxiliary domain, which is always very large in real-world application. As a consequence, the constructed regression problem is very high-dimensional. Below we will propose two methods to improve the learning performance of the constructed regression problem.

Firstly, we can filter out the top *K* rated items from the original rating matrices of the auxiliary domains. All the users on the filtered columns will compose a denser submatrix. Thus we can effectively reduce the dimension of the problem and reserve the user preference as much as possible.

Secondly, it always leads to underfitting or overfitting problem for parametric methods on high-dimensional problem. For example, a linear or quadratic regression model may not fit the high-dimensional data well (leading to underfitting problem), while a high-order regression model may fit the high-dimensional data severely (leading to overfitting problem). It is difficult to choose a proper order or a proper form for the parametric regression models. To overcome the drawback of parametric regression models, we employ a nonparametric regression model, that is, Locally Weighted Linear Regression (LWLR), for the constructed regression problem. Details of LWLR model are given in the following.

Firstly, we expand the constructed feature vector by adding *x*_0_ = 1 (this is the intercept term). Then we build LWLR model in the following form:

(10) min fθ=∑kwkyk−θTxk2,

where

(11) θ=θ0,θ1,…,θnT,

(12) wk=exp⁡−xk−xTxk−x2τ2.

***θ*** denotes the coefficient vector of the linear equation; *w*^(*k*)^ denotes the weight parameter computed by a Gaussian kernel function; *τ* is called the bandwidth parameter determined by cross-validation method [16]; *n* denotes the dimension of the constructed feature vector; *k* denotes the index of training samples; **x**^(*k*)^ denotes the feature vector of the *k*th training samples; *y*^(*k*)^ denotes the corresponding rating value; and **x** is the query point. Thus LWLR is also a lazy learning method.

As LWLR is a nonparametric regression method, it can effectively avoid underfitting or overfitting problem occurring in parametric regression methods. In this paper, we use stochastic gradient descent to solve the optimization problem (10). The update formula of ***θ*** is in the following form, where *a* is the learning rate:

(13) $$\begin{array}{c} \theta_{j}=\theta_{j}+2aw^{\left(i\right)}\left(\;y^{\left(i\right)}-\theta^{T}x^{\left(i\right)}\;\right)x_{j}^{\left(i\right)},\;j=0,\ldots ,n. \end{array}$$

The detailed algorithm is shown in Algorithm 1.

The complete algorithm of FCLWLR is given in Algorithm 2.

## 4. Experiments

In this section, we conduct extensive experiments to test the performance of the proposed algorithm. We compare our algorithm with seven state-of-the-art algorithms, namely, N-CF-U, UVD [6], CFONMTF [17], N-CDCF-U, MF-CDCF, CMF, and CDTF, where N-CF-U, UVD, and CFONMTF are three single-domain CF algorithms and N-CDCF-U, MF-CDCF, CMF, and CDTF are four cross-domain algorithms. All experiments are run on 2.20 GHz, Intel (R) Core (TM) i5-5200U CPU with 8 GB main memory under Windows 7. All algorithms are implemented with Matlab 2015B on top of one open source library for recommender systems, MyMediaLite [18], which implements most common CF approaches.

### 4.1. Data Sets

We conduct this experiment on Amazon product copurchasing network metadata [19] which consists of rating information of users in different domains. 99% of the items belong to 4 main product groups, Books, Music CDs, DVDs, and VHS video tapes. The data set contains 7,593,243 ratings on the scale 1–5 provided by 1,555,170 users over different products, including 393,561 books, 103,144 music CDs, 19,828 DVDs, and 26,132 VHS video tapes. Since the data format of the metadata can be shown in Box 1, it is not suitable to run recommendation algorithm directly. Hence we first convert the data format into a set of triples, (*u*, *i*, *r*), where *r* is the rating of user *u* on item *i*.

### 4.2. The Setting of the Compared Methods

*(1) N-CF-U. *A user-based neighborhood CF model: in this experiment, we use *k* = 10 closest users.

*(2) UVD. *The UV decomposition model: map both users and items to a joint latent factor space of dimensionality *f*. In our experiment, we try different latent factors {5,10,15,20}. The weight of the regularization terms *λ* is tried with different values, {0.001, 0.01, 0.1, 1, 10, 100}. The learning rate *γ* is a constant typically having a value between 0.0 and 1.0. If the learning rate is too small, then learning will occur at a very slow pace. If the learning rate is too large, then oscillation between inadequate solutions may occur. In this paper, for simplicity, we set *γ* = 0.3.

*(3) CFONMTF [17]. *A coclustering based collaborative filtering model using orthogonal nonnegative matrix trifactorization: following the parameter setting method in [17], we compute the optimal value of *λ* and *δ* alternately, where *λ* and *δ* reflect the weights of three different models: ONMTF, user-based, and item-based. In detail, we conducted two experiments on each training set to identify the optimal combination coefficients. Firstly, let *λ* = 0 and compute the optimal value of *δ* which corresponds to the best evaluation metric when *δ* varies from 0 to 1. Secondly, we fix *δ* to be the optimal value and continue to compute the optimal value of *λ*. Besides, we choose 20 as the number of user/item clusters, 30% as the percentage of preselected user/item neighbors, and 20 as the size of user/item neighbors.

*(4) N-CDCF-U. *A cross-domain version of N-CF-U: in this experiment, we use *k* = 10 closest users.

*(5) MF-CDCF. *A cross-domain version of UVD model: here the setting is the same as that of UVD.

*(6) CMF. *This is the collective matrix factorization, which couples rating matrices for all domains on the* User* dimension so as to transfer knowledge through the common user-factor matrix.

*(7) CDTF. *The Cross-Domain Triadic Factorization model: we use the same setting as that in [9]. More specifically, we also take the following strategy to initialize the individuals with exponential growth, where *a* ∈ (0,1] is a constant to scale weight, *β* and *γ* are integers to control the range of weight, and 1 is an all-one vector with the length equal to the number of auxiliary domains

(14) $$\begin{array}{c} w_{i}^{a}=\left(\;a\times 10^{i}\;\right)\times 1\;i=\beta ,\ldots ,\gamma . \end{array}$$

In this experiment, to find the optimal weights assignment, we ran the GA with initial population **w** = {**w**^0.33^, **w**^0.66^, **w**^1^} and *β* = −2, *γ* = 2; that is, there are totally 15 initial individuals with different scale.

*(8) FCLWLR. *The proposed method using rating data from all the auxiliary domains: in this experiment, for the rating filling process with N-CF-U algorithm, we also use *k* = 10 closest users. Different bandwidth parameters *τ*^2^ ∈ {0.125,0.25,0.5,1, 2,4, 8} are tried and we use cross-validation method to compute the best parameter value. For simplicity, we also set the learning rate *a* = 0.3.

*(9) FCLWLR_CD. *This is the proposed method only using rating data from the auxiliary domain of Music CDs.

*(10) FCLWLR_DVD. *This is the proposed method only using rating data from the auxiliary domain of DVDs.

*(11) FCLWLR_VHS. *This is the proposed method only using rating data from the auxiliary domain of VHS video tapes.

### 4.3. Evaluation Protocol

We first use mean absolute error (MAE) as an evaluation metric in our experiments. MAE is defined as

(15) $$\begin{array}{c} \frac{\left(\sum_{i\in T}\left|r_{i}-{\tilde{r}}_{i}\right|\right)}{\left|T\right|}, \end{array}$$

where *T* denotes the set of test ratings, *r*_*i*_ is the ground truth, and ${\tilde{r}}_{i}$ is the predicted rating. A smaller value of MAE means a better performance.

However, what we often want is not to make a rating prediction for any item but to find the best items. In Top*N* recommendations, a recommender is trying to pick the best *N* items for someone. Hence, a model with a smaller value of MAE does not mean a better recommendation performance. Rather than getting the exact rating right, in Top*N* recommendations we are interested in predicting whether an item would be among the user's favorites.

In our experiments, we also use another two metrics commonly used in information retrieval, that is, precision and recall, to measure the recommendation quality. Let *R*(*u*) be a recommended list based on the behavior of the user on the training set, and *T*(*u*) is the “liked” list of behaviors that the user has on the test set. Then, the precision of the recommended results is defined as

(16) $$\begin{array}{c} precision=\frac{\sum_{u\in U}\left|R\left(u\right)\cap T\left(u\right)\right|}{\sum_{u\in U}\left|R\left(u\right)\right|}. \end{array}$$

The recall of the recommended results is defined as

(17) $$\begin{array}{c} recall=\frac{\sum_{u\in U}\left|R\left(u\right)\cap T\left(u\right)\right|}{\sum_{u\in U}\left|T\left(u\right)\right|}. \end{array}$$

Precision gives us an estimate of how many of the items predicted to be “liked” for a user really belong to the “liked” list. Recall estimates how many of all the items in the user's “liked” list were predicted correctly.

### 4.4. Data Preparation for MAE

We construct two data sets to conduct the experiment. In one data set, we selected Books as the target domain and Music CDs, DVDs, and VHS video tapes as the auxiliary domains. In the other data set, we selected Music CDs as the target domain and Books, DVDs, and VHS video tapes as the auxiliary domains.

For the first data set, we filtered out users who have rated at least 30 music CDs, 30 DVDs, and 30 VHS video tapes so as to construct denser rating matrices in the auxiliary domains. Finally, 496 users were selected, and in addition we retrieved all items rated by these users in these four domains and set aside top *K* rated items for each domain, respectively. Thus the submatrices in the auxiliary domains are much denser than the original rating matrices.  Table 1 shows the statistics of the data set for evaluation.

For the second data set, we filtered out users who have rated at least 90 Books, 30 DVDs, and 30 VHS video tapes so as to construct denser rating matrices in the auxiliary domains. Finally, 435 users were selected, and in addition we also retrieved all items rated by these users in these four domains and set aside top *K* rated items for each domain, respectively.  Table 2 shows the statistics of the data set for evaluation.

To simulate the sparse data problem, we constructed two sparse training sets, tr_20_ and tr_75_, by, respectively, holding out 80% and 25% data from the target domain Book; that is, the remaining data of target domain for training is 20% and 75%. The hold-out data serve as ground truth for testing. Likewise, we also construct two other training sets tr_20_ and tr_75_ when choosing Music as the target domain.

### 4.5. Data Preparation for Precision and Recall

We choose Books as the target domain and Music CDs, DVDs, and VHS video tapes domains as the auxiliary domains. We filtered out users who have rated at least 100 books so that there are enough observations to be split in various proportions of training and testing data for our evaluation. Finally, 586 users were selected, and in addition we retrieved all items rated by these users in the four domains and set aside top *h* rated items for each domain, respectively.  Table 3 shows the statistics of the filtered data. Then, we constructed rating matrices over filtered data for each domain.

To simulate the sparse data problem, we constructed five sparse training sets, TR_50_, TR_40_, TR_30_, TR_20_, and TR_10_, by, respectively, holding out 50%, 60%, 70%, 80%, and 90% rating data from the target domain Book; that is, the remaining data for training is 50%, 40%, 30%, 20%, and 10%. The testing set is constructed in the following. We first filtered out users who have rated more than 20 books from the set composed by the hold-out data. Then we select 20 books randomly for each filtered user as the testing set. In order to compute the precision and recall, for the testing set, we also map the five classes of original ratings {1,2, 3,4, 5} into 2 classes, “liked” and “disliked.” Usually, an item with a score greater than or equal to 3 is defined as “liked”; otherwise, it is defined as “disliked.”

We define the size of the recommendation list *N* = 3,6, 9 and the set of all the books liked by the user from the 20 books as the “liked” list. We sort the predictive ratings of the 20 books for each user in the testing set and choose the top *N* books for recommendation. The books in the recommendation list are labeled as “liked.” Hence, we can compute the precision and recall.

### 4.6. Impact of Rating Densities in Auxiliary Domains

FCLWLR requires that auxiliary domains contain dense rating data. Obviously, a very sparse rating matrix from an auxiliary domain will not improve the recommendation performance in the target domain. In this part, we analyze how the performance of FCLWLR is affected by rating densities in auxiliary domains. We construct the experiment data in the following way.

For simplicity, we just use tr_20_ as the training data in the target domain. For data in auxiliary domains, we constructed four different data sets, *d*_100_, *d*_75_, *d*_50_, and *d*_25_, by, respectively, holding out 0%, 25%, 50%, and 75% rating data. We use MAE to evaluate the performance of FCLWLR on different data sets.

### 4.7. Results

**Figure 4:**
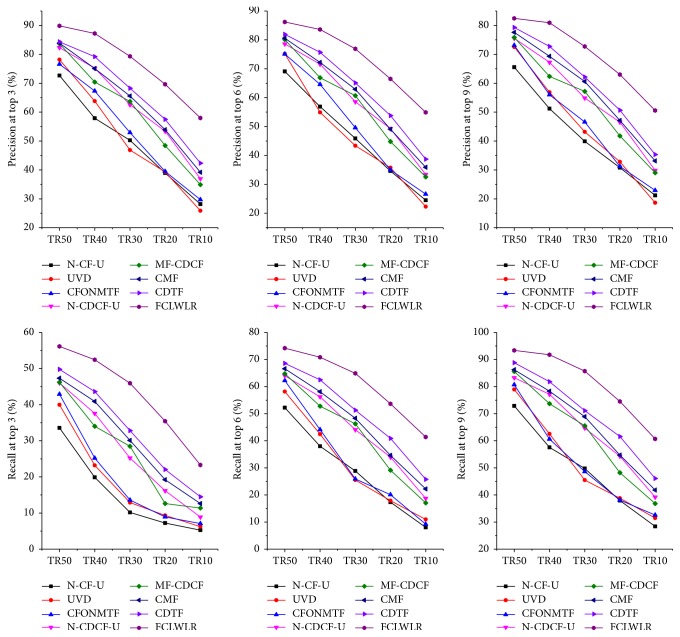
The precision and recall results for some algorithms.

**Figure 5:**
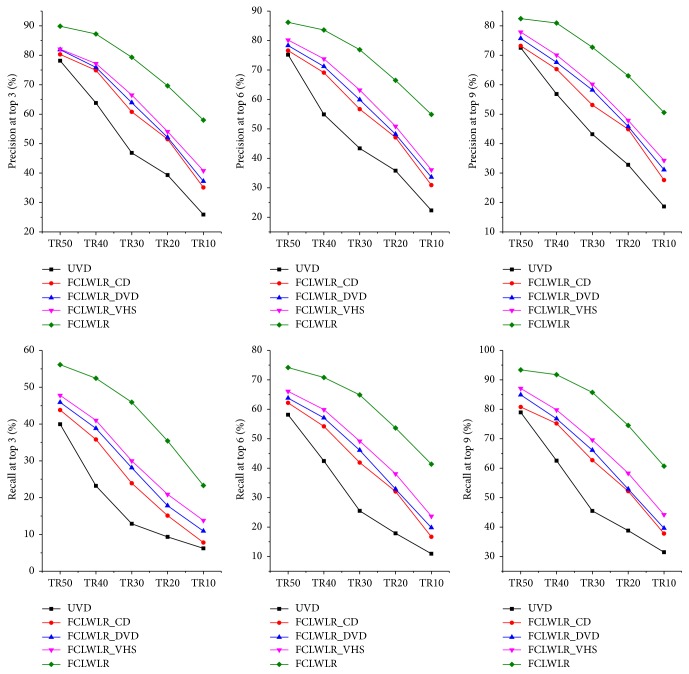
The precision and recall results for other algorithms.

**Figure 6:**
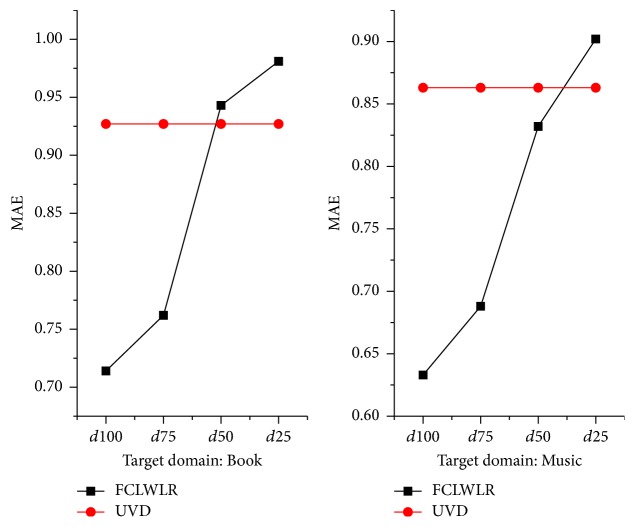
MAE scores of FCLWLR on different data sets.

The comparison results of different algorithms on MAE and on precision and recall are reported in Tables 4 and 5 and Figures 4 and 5, respectively. The performances of FCLWLR on different data sets are given in Figure 6.

As shown in Table 4 and Figure 4, the five CDCF models (N-CDCF-U, MF-CDCF, CMF, CDTF, and FCLWLR) all perform better than N-CF-U, UVD, and CFONMTF under three different evaluation metrics, because N-CF-U, UVD, and CFONMTF are single-domain CF algorithms which cannot deal with the sparsity problem effectively. The performances of N-CDCF-U and MF-CDCF are roughly equal. Since both N-CDCF-U and MF-CDCF fail to consider the differences among domains, as expected they perform worse than the other three models (CMF, CDTF, and FCLWLR) which take this difference into account. Our model FCLWLR performs much better than CMF and CDTF. This is due to the fact that our model uses different feature sets to represent different domains, which avoids computing proper weights to different domains. As for how to compute proper weights for different feature, many sophisticated supervised learning models can help us. Our model uses LWLR model to compute proper weights for different features. Since LWLR is a nonparametric regression method, it can effectively avoid underfitting or overfitting problem occurring in parametric regression methods. Hence it can guarantee the accuracy of the weights. However, CMF does not provide a mechanism to find an optimal weights assignment for the auxiliary domains. Though CDTF assigns the weights based on genetic algorithm (GA), the performance is susceptible to the setting of the initial population.

As shown in Table 5 and Figure 5, the proposed models using rating data from one auxiliary domain or from all the auxiliary domains all perform better than UVD model which just uses the rating data from the target domain. FCLWLR_DVD and FCLWLR_VSH outperform FCLWLR_CD due to the fact that DVDs and VHS video tapes are more related to books than music, since many movies are adapted from novels, and movies and books have some correspondence in genre. Besides, FCLWLR perform best due to the fact that more user features can be considered in the regression model, which will improve the regression performance effectively.

According to Table 4, it is also worth noting that, from tr_20_ to tr_75_, our method possesses the largest performance improvements, because, with the number of training ratings increasing, the training set size of the converted regression problem also increases. Thus the regression model can effectively avoid overfitting, and the performance can be improved. N-CDCF-U also achieves a not-bad performance when the data is relatively dense, that is, tr_75_, but the performance decreases very fast when the data becomes sparser, because when the data are sparse, the total similarity used in N-CDCF-U cannot represent the local similarity in the target domain well. However, according to (1), with the number of training ratings increasing, the total similarity can represent the local similarity in the target domain better.

From Figures 4 and 5, we can also obtain two important conclusions in the following.

(1) Precision and recall metrics always depend on the length of the recommended list *N*. In general, as *N* increases, the precision metric will decrease and the recall metric will increase.

(2) If *N* < *l*, where *l* denotes the length of the “liked” list, the recall metric for any model will not be greater than *N*/*l*.

We have the following observations from Figure 6. (1) When rating matrices of auxiliary domains are relatively dense (e.g., *d*_100_, *d*_75_), our model FCLWLR performs well. The effect of FCLWLR, however, is unsatisfactory when rating matrices of auxiliary domains are sparse (e.g., *d*_50_, *d*_25_). (2) FCLWLR even performs worse than UVD that is a single-domain CF algorithm, when rating matrices of auxiliary domains are very sparse. The main reason may be that when rating matrices become sparser, noise data from auxiliary domains will have a worse impact on the recommendation performance in the target domain.

## 5. Conclusion

In this paper, from the perspective of regression, we propose a cross-domain collaborative filtering algorithm based on Feature Construction and Locally Weighted Linear Regression (FCLWLR). On one side, the FCLWLR model can avoid computing proper weights for different domains, since we construct features in each domain and use the features to represent the domains. On the other side, the FCLWLR model can guarantee the accuracy of the weights for different features, due to the fact that LWLR model is a nonparametric regression method, which can effectively avoid underfitting or overfitting problem. The experimental results have shown that FCLWLR can significantly outperform all other state-of-the-art baseline algorithms at various sparsity levels.

In this paper, we have only discussed how to construct features about users with the help of rating information in the auxiliary domains. In our future work, we will explore how to construct features about items and how to combine both user and item features to provide a better recommendation. Besides, FCLWLR requires a relatively rich rating data from the auxiliary domain. The experimental results show that FCLWLR even performs worse than single-domain CF algorithms, when rating matrices of auxiliary domains are very sparse. It would be interesting to compute what sparsity of rating data from auxiliary domains will degrade the effectiveness of FCLWR. It is worth studying in our future work.

## Figures and Tables

**Box 1:**
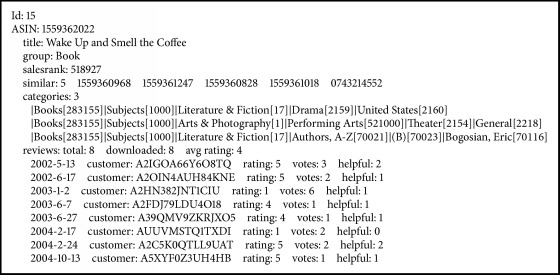
Amazon metadata format.

**Algorithm 1:**
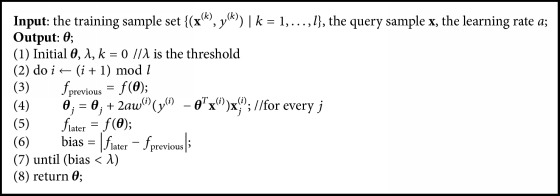
The stochastic gradient descent algorithm for LWLR model.

**Algorithm 2:**
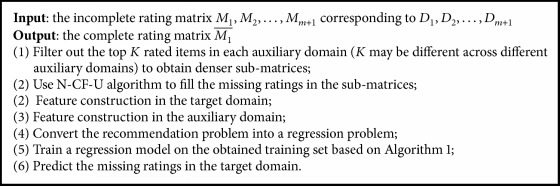
The FCLWLR algorithm.

**Table 1:** Statistics of the first data set for evaluation.

Domain	*K*	Avg. # of ratings for each item	Avg. # of ratings for each user	Density
**Books**	100	29.89	6.03	6.03%
*Music CDs*	100	34.65	6.99	6.99%
*DVDs*	100	61.77	12.45	12.45%
*VHS video tapes*	100	59.43	11.98	11.98%

**Table 2:** Statistics of the first data set for evaluation.

Domain	*K*	Avg. # of ratings for each item	Avg. # of ratings for each user	Density
**Music CDs**	100	50.03	11.5	11.50%
*Books*	100	62.74	14.42	14.42%
*DVDs*	100	59.12	13.59	13.59%
*VHS video tapes*	100	50.03	11.5	11.50%

**Table 3:** Statistics of the filtered Amazon data.

Domain	*h*	Avg. # of ratings for each item	Avg. # of ratings for each user	Density
**Books**	500	41.37	35.30	7.06%
*Music CDs*	500	47.58	40.60	8.12%
*DVDs*	500	70.03	59.75	11.95%
*VHS video tapes*	500	60.12	51.30	10.26%

**Table 4:** MAE scores for some algorithms.

Methods	Target domain: Book		Target domain: Music	
	tr_75_	tr_20_	tr_75_	tr_20_
N-CF-U	0.756	0.947	0.650	0.879
UVD	0.727	0.927	0.597	0.863
CFONMTF	0.720	0.919	0.592	0.866
N-CDCF-U	0.680	0.906	0.541	0.846
MF-CDCF	0.692	0.902	0.566	0.839
CMF	0.679	0.789	0.506	0.737
CDTF	0.652	0.745	0.489	0.649
*FCLWLR*	*0.475*	*0.714*	*0.282*	*0.633*

**Table 5:** MAE scores for other algorithms.

Methods	Target domain: Book	
	tr_75_	tr_20_
UVD	0.727	0.927
FCLWLR_CD	0.692	0.913
FCLWLR_DVD	0.686	0.803
FCLWLR_VHS	0.665	0.761
*FCLWLR*	*0.475*	*0.714*
